# The American Plan of Hospital Service

**Published:** 1921-04-02

**Authors:** 


### The American Plan of Hospital Service.

### PURPOSE AND SCOPE OF THE LIBRARY.

The proposed library and service bureau will collect, classify for reference use, and distribute types of data as outlined below. Pamphlets and data will also be collected and filed in such form as to be readily available to make up bundles to be sent out in answer to inquiries.

(1) Plans, drawings, and other data pertaining to the construction of hospitals, dispensaries, first-aid rooms, etc. Also follow-up of all new hospitals within one year of their opening for the purpose of appraising efficiency and adaptability of architectural arrangement.

(2) Complete record of hospital architects with lists of hospitals planned by them.

(3) Records of equipment in new hospitals, dispensaries, etc., and a follow-up for the purpose of ascertaining what part of the equipment proved unnecessary and what additional equipment was found necessary.

(4) Indexes of hospital supplies and equipment, and equipment necessary for certain work with cost estimates.

(5) Case record systems with discussions and comparative data.

(6) Health and hospital literature and reference material on community problems, vital statistics, social service, public health nursing, legal subjects, new laws, and pending legislation affecting hospitals.

(7) Material and data concerning preliminary educational and publicity work incident to the promotion of hospitals. Data on preliminary work incident to the promotion of hospitals. Data on preliminary and permanent organisation of hospital boards and information regarding methods of business organisation and financing.

(8) Lists of names of suitable and desirable persons with the records of their work will be kept available for those desiring to employ persons for special work, as for various surveys, campaigns, etc., and for expert advice on various subjects.

(9) Complete records of all organisations and associations in the hospital health field, with names of officials, information as to purposes, scope, and places of meeting.

(10) Information as to internal organisation and management and function and work of the various departments.

Clientele to be served:

(1) Hospital, medical, nursing, and health organisations and publications; and the trustees, organisers, and proprietors of these.

(2) Hospital organisers, trustees, superintendents, medical staff members, department heads, and other executives in official capacity or as individuals.

(3) Building committees and committees organised for the promotion of a hospital project.

(4) Directors of dispensaries and first-aid workers in industries, schools, and colleges.

(5) Architects.

(6) Public officials.

(7) Others having practical needs.

